# Efficacy of therapeutically administered gepotidacin in a rabbit model of inhalational anthrax

**DOI:** 10.1128/aac.01497-23

**Published:** 2024-02-15

**Authors:** Jamese J. Hilliard, Charles Jakielaszek, Frank Mannino, Mohammad Hossain, Lian Qian, Cindy Fishman, Samandra Demons, Jeremy Hershfield, Carl Soffler, Riccardo Russo, Lisa Henning, Joseph Novak, Karen O'Dwyer

**Affiliations:** 1 GSK, Collegeville, Pennsylvania, USA; 2 US Army Medical Research Institute of Infectious Diseases, Fort Detrick, Maryland, USA; 3 Rutgers University School of Medicine, Newark, New Jersey, USA; 4 Battelle Biomedical Research Center (BBRC), Columbus, Ohio, USA; Providence Portland Med Ctr, Portland, Oregon, USA

**Keywords:** *Bacillus anthracis*, biothreat, gepotidacin, FDA Animal Rule

## Abstract

*Bacillus anthracis* is a Gram-positive Centers for Disease Control and Prevention category “A” biothreat pathogen. Without early treatment, inhalation of anthrax spores with progression to inhalational anthrax disease is associated with high fatality rates. Gepotidacin is a novel first-in-class triazaacenaphthylene antibiotic that inhibits bacterial DNA replication by a distinct mechanism of action and is being evaluated for use against biothreat and conventional pathogens. Gepotidacin selectively inhibits bacterial DNA replication via a unique binding mode and has *in vitro* activity against a collection of *B. anthracis* isolates including antibacterial-resistant strains, with the MIC_90_ ranging from 0.5 to 1 µg/mL. *In vivo* activity of gepotidacin was also evaluated in the New Zealand White rabbit model of inhalational anthrax. The primary endpoint was survival, with survival duration and bacterial clearance as secondary endpoints. The trigger for treatment was the presence of anthrax protective antigen in serum. New Zealand White rabbits were dosed intravenously for 5 days with saline or gepotidacin at 114 mg/kg/d to simulate a dosing regimen of 1,000 mg intravenous (i.v.) three times a day (TID) in humans. Gepotidacin provided a survival benefit compared to saline control, with 91% survival (*P*-value: 0.0001). All control animals succumbed to anthrax and were found to be blood- and organ culture-positive for *B. anthracis*. The novel mode of action, *in vitro* microbiology, preclinical safety, and animal model efficacy data, which were generated in line with Food and Drug Administration Animal Rule, support gepotidacin as a potential treatment for anthrax in an emergency biothreat situation.

## INTRODUCTION

*Bacillus anthracis,* the etiologic agent of anthrax, is a Gram-positive spore-forming rod-shaped bacterium that is often found in the soil and commonly affects domestic and wild animals. Humans are incidentally infected through contact with infected animals or animal products. The major forms of anthrax are cutaneous, inhalational, gastrointestinal, and injectional. Cutaneous anthrax is the most frequent form of the disease and, although it can be fatal in 20% of cases, in the majority of the cases, it is self-limiting (1, 2). Inhalation anthrax is the most aggressive and lethal form of the disease, with a reported incubation period of 4–6 days from exposure to initial clinical symptoms and death; thus, treatment should be initiated as soon as possible (2). The bacterial spore is the infectious form leading to disease. The major virulence factors are the poly-γ-D-glutamic acid capsule, which is resistant to both phagocytosis and complement activation, and a tripartite toxin, which is composed of a receptor-binding subunit, protective antigen (PA), two enzymatic subunits, lethal factor (LF), a zinc metalloprotease and edema factor (EF), and a calmodulin-dependent adenylate cyclase. After binding to the receptor on the host’s cell surface, the PA is cleaved, resulting in the exposure of binding sites for which LF and EF compete. The PA forms a membrane-inserting heptamer, which translocates LF or EF into the cell. These subunits interact to form the active toxins: lethal toxin (PA +LF) and edema toxin (PA +EF). At the initial stages of infection, lethal toxin (LT) and edema toxin (ET) coordinately impair the host’s innate immune response, enabling the pathogen to establish infection. When elevated toxin concentrations are reached, LT and ET can directly cause host death by targeting both the cardiovascular system and the liver (3–5). Without aggressive prophylaxis or intervention, inhalational anthrax can result in mortality rates approaching 90% (6–8). Fatal anthrax is ultimately the result of acute toxemia, massive bacteremia, and the host’s response to the toxins and bacteria (1–3). The importance of anthrax toxins during the latter stages of disease becomes apparent as patients can still die from a systemic infection after bacteria are no longer detectable (3).

High-titer anthrax spores can be easily generated using basic microbiological techniques, and the ability of these spores to be rapidly disseminated by aerosolization has made anthrax a bio-weapon and military threat. An anthrax outbreak in 1979 in Sverdlosk, Russia, and the 2001 attacks in the United States (US) illustrate that inhalational anthrax can be rapidly fatal (6, 8, 9). Following the 2001 civilian attacks in the US, an emphasis on post-exposure therapeutics has become a research priority (10–12).

Gepotidacin is a novel first-in class bactericidal triazaacenaphthylene antibiotic. It was discovered by GSK, with further development supported by a public–private partnership between GSK, the Defense Threat Reduction Agency (DTRA, U.S. Department of Defense), and the Biomedical Advanced Research and Development Authority (U.S. Department of Health and Human Services), and it is being evaluated for use against both biothreat and conventional pathogens. Gepotidacin inhibits bacterial DNA replication by a distinct mechanism of action (13–15), demonstrating *in vitro* and *in vivo* activity against a range of Gram-positive and Gram-negative bacterial pathogens (16–22). Structural models of bacterial type II topoisomerase enzymes reveal the novel binding mode of this class of antibacterials and distinguish it from the binding mode of the fluoroquinolones (13, 15). As a consequence of this novel mode of action, *in vitro* activity is maintained against most target pathogens carrying resistance determinants to other antibacterials, including fluoroquinolones (16, 17). Gepotidacin is currently being evaluated for investigational oral treatment of uncomplicated urinary tract infection and uncomplicated gonorrhea, including those caused by pathogens resistant to currently used antimicrobials, (www.clinicaltrials.gov: NCT04020341, NCT04010539, and NCT04187144). In addition, gepotidacin has the potential to treat bacterial biothreat infections, including anthrax (23, 24).

It is neither practical nor ethical to conduct inhalational anthrax clinical trials in humans. Between 1900 and 1976, only 17 cases of inhalational anthrax were reported in the US, with an additional 11 cases reported in the 2001 anthrax attacks (25). Under the Food and Drug Administration’s (FDA’s) Animal Rule, establishing efficacy in well-characterized animal models is essential for the development of therapeutics directed against anthrax (26, 27). The New Zealand White (NZW) rabbit has been shown to be an appropriate model for inhalational anthrax, and the physiologic changes following aerosol challenge with *B. anthracis* in the rabbit have demonstrated that both the manifestations of the disease and the pathology were similar to those observed in humans, though the disease progresses more rapidly in rabbits. Furthermore, rabbits are predictive of the outcome of inhalational anthrax in nonhuman primate infection models. Refinements of the model have incorporated the use of biomarkers, such as a significant increase in body temperature (SIBT) and the presence of circulating PA, as triggers for therapeutic intervention (8, 12, 28, 29).

Gepotidacin has demonstrated *in vitro* activity against *B. anthracis* and efficacy in proof-of-concept rabbit inhalational challenge models (20). It was therefore of interest to evaluate gepotidacin in a study designed to provide pivotal efficacy data under the FDA Animal Rule and to predict effective clinical dosing regimens for gepotidacin in the treatment of inhalational anthrax.

## RESULTS

### Gepotidacin demonstrates *in vitro* activity against *B. anthracis*

A total of 160 isolates of *B. anthracis* from the U.S. Army Medical Research Institute of Infectious Diseases (USAMRIID) (Study 1, *n* = 30; Study 2, *n* = 100) and Rutgers University (*n* = 30) were tested to evaluate the *in vitro* activity of gepotidacin (Table 1). The MIC_90_ values for gepotidacin ranged from 0.5 µg/mL to 1 µg/mL. Table 2 shows the activity of gepotidacin against the plasmid-cured *B. anthracis* Ames strain (ΔANR) and its isogenic mutants, S1-1 and S1-2. These single-step mutants have modifications in DNA gyrase at the following positions: S85L (S1-1) and E89K (S1-2). The MICs for the S1-1 and the S1-2 mutants increased 4- to 32-fold for levofloxacin and ciprofloxacin, respectively, compared to the parent strain. In contrast, there was only a modest 2-fold increase in the gepotidacin MIC for both mutants.

**TABLE 1:** Gepotidacin MIC values for a collection of *B. anthracis* strains (*N* = 160)^*a*^

	Number of isolates	MIC range	MIC_50_	MIC_90_
		(μg/mL)	(μg/mL)	(μg/mL)
USAMRIID diversity set	30	0.5–2	1	1
USAMRIID collection	100	0.12–1	0.5	0.5
Rutgers	30	0.25–1	0.5	1

^*a*^ MIC, minimum inhibitory concentration; USAMRIID, United States Army Medical Research Institute of Infectious Diseases.

**TABLE 2:** Activity of gepotidacin, ciprofloxacin, and levofloxacin against attenuated *B. anthracis* single-step gyrase mutants^*c*^

Strain	Amino acid change	Frequency of isolation^*b*^	Gepotidacin	Ciprofloxacin	Levofloxacin
			MIC	MIC	MIC
			(μg/mL)	(μg/mL)	(μg/mL)
Parent ΔANR^*a*^	NA	NA	1	0.03	0.06
S1-1^	S85L	80%	2	1	0.25
S1-2^	E89K	20%	2	1	0.25

^*a*^ Plasmid-cured *B. anthracis* Ames strain.

^*b*^ Laboratory-generated mutants; the frequency of isolation is from the original publication (30).

^*c*^ MIC: minimum inhibitory concentration; NA: not applicable.

### Therapeutically administered gepotidacin enhances survival and reduces disease severity

NZW rabbits challenged with a targeted dose of 200 LD_50_ *B. anthracis* Ames spores received a mean inhalation exposure of 191 LD_50_ equivalents (range 106–258) for all study animals. All rabbits were negative for the PA electrochemiluminescence (PA-ECL) assay prior to challenge, and the median time for a positive PA-ECL post challenge was 28 h for both gepotidacin and saline groups. The time to trigger (positive PA-ECL) was comparable between groups (Table 3). All animals were bacteremic following trigger and prior to treatment, with an average bacterial burden of 3.0 Log_10_ colony forming unit (CFU)/mL in the blood. The median time to bacteremia was 25 h following challenge and was comparable between treatment groups: 24.7 h vs 25.5 h for gepotidacin and saline groups, respectively. No relationship between inhaled spore exposure and time to trigger was demonstrated. The median time to a significant increase in body temperature was comparable between the gepotidacin- and saline-treated groups: 29.3 h and 27.9 h, respectively (Table 3).

**TABLE 3:** Aerosol exposure range, time to trigger, time to treatment, bacteremia onset, and challenge dose^*b*^^,^^*c*^

Treatment	LD_50_ exposure average (range)	Time to trigger^*a*^ (hours post challenge) median (95%)	Time to onset of bacteremia (hours) median (95%)	% Bacteremic PTT	Time to SIBT median (95%)
Gepotidacin	180 (106–257)	28.4 (24.5–35.4)	24.7 (22.6–28.6)	100	29.3 (25.2–30.6)
Saline	212 (157–258)	28.3 (23.3–38.3)	25.5 (23.0–31.3)	100	27.9 (23.9–33.4)

^*a*^ Trigger = positive PA-ECL.

^*b*^ No statistical difference between the LD_50_ exposure, time from trigger to treatment, bacteremia onset, or challenge dose (two-tailed *t*-test).

^*c*^ LD_50_, lethal dose; PTT = prior to treatment; SIBT = significant increase in body temperature.

**Fig 1:**
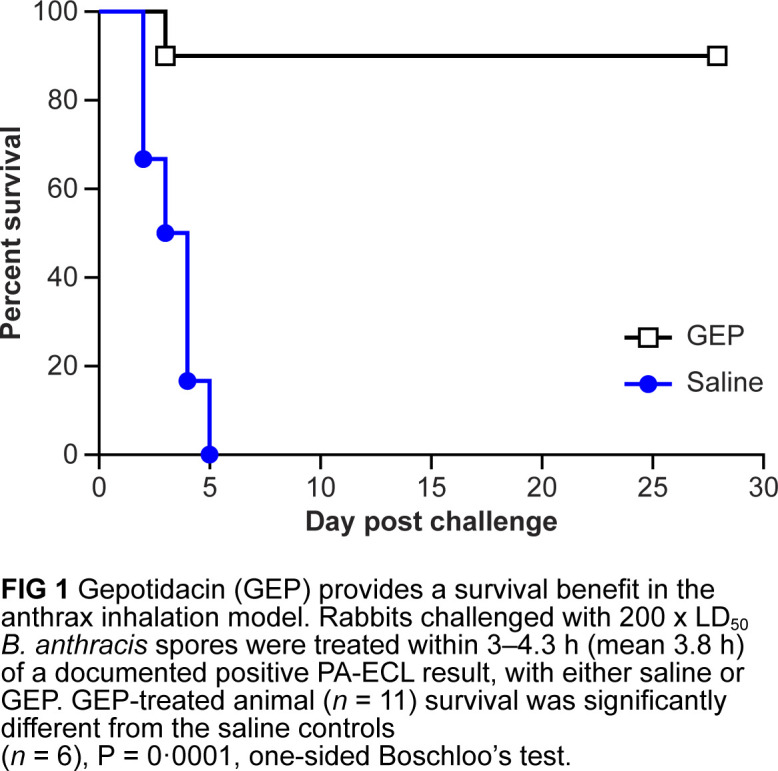
Gepotidacin (GEP) provided a survival benefit in the anthrax inhalation model. Rabbits challenged with 200 x LD_50_ *B. anthracis* spores were treated within 3 to 4.3 h (mean 3.8 h) of a documented positive protective antigen electrochemiluminescence result, with either saline or GEP. The survival of GEP-treated animals (*n* = 11) was significantly different from that of the saline control (*n* = 6), *P* = 0.0001, one-sided Boschloo’s test.

Gepotidacin was efficacious and provided a clear survival benefit compared to saline control (one-sided Boschloo’s test, *P* < 0.0001). As shown in Fig. 1, all six saline-treated animals succumbed 2 to 5 days post-challenge. Three animals died prior to euthanasia, and three were euthanized based on pre-defined euthanasia criteria. In contrast, 10 of the 11 (90.1%) gepotidacin-treated rabbits survived until the end of the study (day 28 post-challenge). The single animal that succumbed (euthanized) on day 3 post-challenge had catheter complications that impacted infusion start times and gepotidacin exposures (e.g., AUC, Cmax, and Tmax); in particular, the first dose exposure was approximately 10% of the AUC values noted for the other treated animals.

### Gepotidacin treatment resolves bacteremia

Terminal blood cultures for all six saline–control animals were positive with high numbers of bacteria, (geometric mean 8.07 log_10_ CFU/mL), and all control animals demonstrated positive organ cultures at necropsy. In contrast, blood and tissues from all surviving animals and animals that succumbed during gepotidacin treatment were cultured for the presence of *B. anthracis,* and all survivors were blood culture-negative by day 7 post challenge. Blood cultures remained negative until the end of the study, i.e., day 28 post challenge. In addition, all organs from surviving gepotidacin-treated animals were culture-negative, with the exception of one animal that was positive for lung and one for spleen with colony-forming units below the limit that could be quantified by the methods used, likely representing latent spores. (Tables 4 and 5). The terminal blood culture from the single gepotidacin-treated animal that had catheter complications with reduced gepotidacin exposure and succumbed was positive; however, the numbers of bacteria were lower relative to those in controls (3.18 log_10_ CFU/mL compared to 8.07 log_10_ CFU/mL). Additionally, organ culture bacterial counts tended to be lower in this animal relative to the saline controls, with the exception of the brain, which had bacterial counts comparable to those in controls.

**TABLE 4:** Geometric means and range for positive quantitative *B. anthracis* bacteremia (log_10_ CFU/mL)^*a*^^,*c*,*d*^

	Gepotidacin		Saline	
Time point	No. of culture-positive/total no. of animals	Geometric mean (range)	No. of culture positive/total no. of animals	Geometric mean (range)
24 h PC	6/11	2.63 (<2.40, 3.04)	3/6	2.88 (<2.40, 3.52)
30 h PC	6/7	2.78 (<2.40, 3.06)	4/4	2.62 (<2.40, 3.17)
36 h PC	3/3	2.66 (<2.40, 3.11)	2/2	2.83 (2.67, 3.0)
42 h PC	1/1	<2.40 (--)	0/0	--(--)
PTT	9/9^*b*^	3.04 (<2.40, 3.82)	6/6	3.30 (<2.40, 5.12)
24 PTI	9/11	3.70 (<2.40, 4.64)	5/5	4.56 (4.05, 6.13)
Day 7 PC	0/10	--(--)	0/0	--(--)
Day 14 PC	0/10	--(--)	0/0	--(--)
Day 28 PC	0/10	--(--)	0/0	--(--)
Terminal	1/1	3.18 (--)	6/6	8.07 (6.15, 8.78)

^*a*^ Parameter was log_10_-transformed for the analysis.

^*b*^ Blood samples were not available for two animals that were positive at time points prior to PTT.

^*c*^ -- Samples were not collected for this group at this time point, or all results were below the LLOQ, or there was a single observation for this group at this time point.

^*d*^ LLOQ = 2.40 log10 CFU/mL; LLOQ = lower limit of quantification; PC = post-challenge; PTT = prior to treatment ; PT1 = post first treatment.

**TABLE 5:** Geometric means and range for positive *B. anthracis* tissue burden (log_10_ CFU/g)^*a*^^,*c*,*d*^

	Gepotidacin		Saline	
Tissue	No. of culture-positive/total no. of animals	Geometric mean (range)	No. of culture-positive/total no. of animals	Geometric mean (range)
Heart	1/11	<3.40 (--)	6/6	7.54 (<3.40, 7.94)
Brain	1/11	6.84 (--)	6/6	6.64 (3.68, 8.27)
Lung	2/11^*b*^	<3.40 (<3.40)	6/6	8.74 (4.49, 9.03)
Kidney	1/11	3.44 (--)	6/6	6.34 (<3.40, 8.15)
Spleen	2/10^*b*^	3.89 (<3.40, 4.22)	6/6	8.31 (6.92, 9.20)
Mediastinal lymph node	1/11	3.44 (--)	6/6	7.75 (7.02, 8.48)

^*a*^ Parameter was log_10_-transformed for the analysis. LLOQ = 3.40 log_10_ CFU/mL (triple plating was used for counts and <25 colonies were seen on two of three plates).

^*b*^ Includes a gepotidacin-treated survivor that was culture-positive at the study end.

^*c*^ -- Range of counts could not be determined since there was only one positive observation for this group at this time point or results were the same.

^*d*^ CFU, colony-forming units; LLOQ, lower limit of quantification.

Gepotidacin MICs were determined for isolates recovered from animals treated with gepotidacin and compared to those of the parent challenge strain to evaluate any development of resistance on therapy. All MICs were the same or within 1 dilution of the parent challenge strain, suggesting no development of resistance in this study.

### Gepotidacin treatment resolves fever

**Fig 2:**
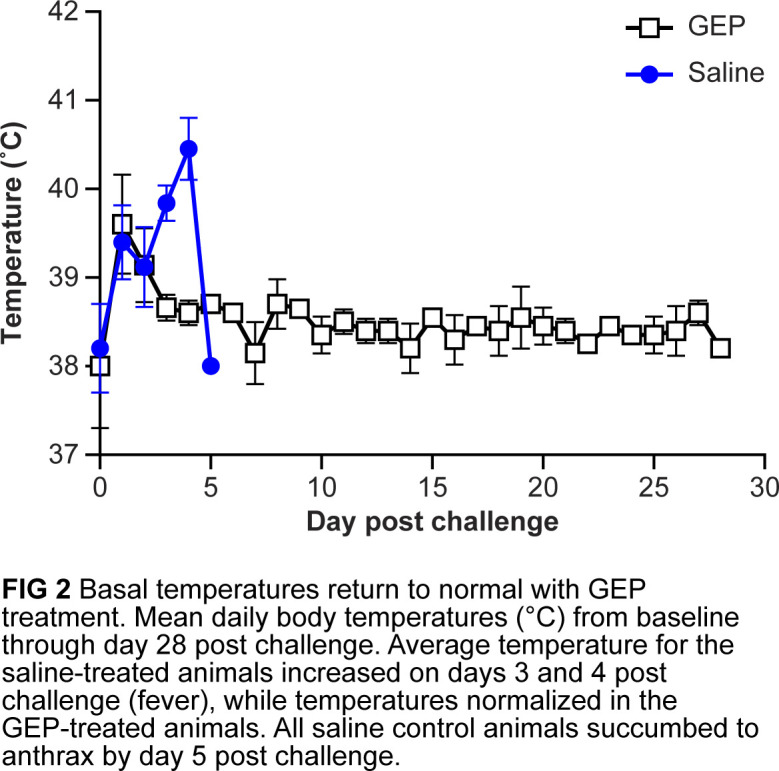
Basal temperatures returned to normal with gepotidacin (GEP) treatment. Mean daily body temperature (°C) from baseline through day 28 post-challenge. Average temperature for the saline-treated animals increased on days 3 and 4 post-challenge (fever), while temperatures normalized in the GEP-treated animals. All saline control animals succumbed to anthrax by day 5 post-challenge.

The average baseline body temperature in the rabbit was 38.0 ± 0.7°C. After challenge, the mean body temperature for both groups of animals increased 1 to 2°C compared to that at baseline and resolved by day 7 post-challenge in the gepotidacin-treated group. The mean body temperature did not return to the baseline level for the saline–control group (Fig. 2).

### Gepotidacin-treated animals develop immune response to LT

**Fig 3:**
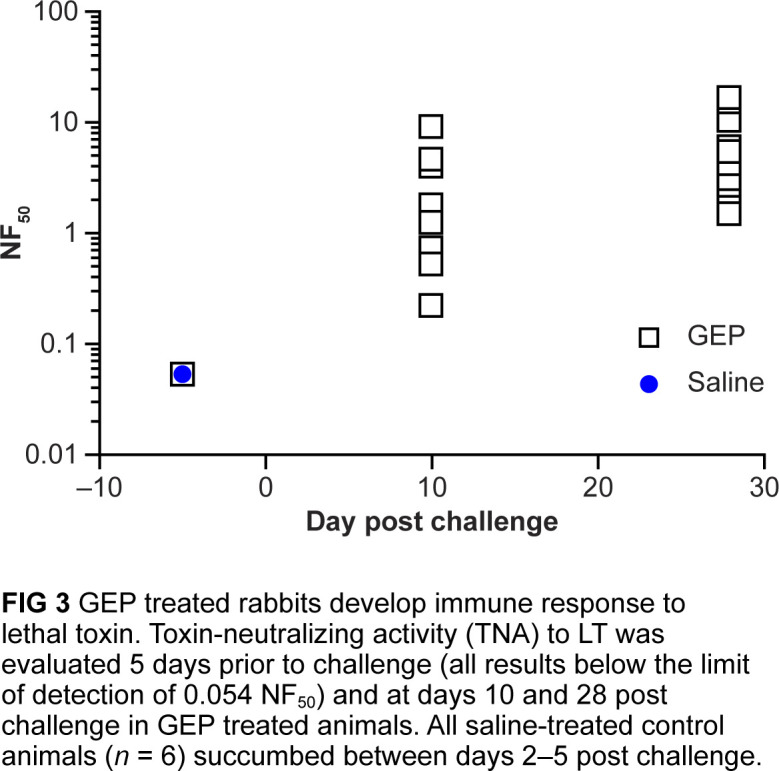
Gepotidacin (GEP)-treated rabbits developed an immune response to lethal toxin. Toxin-neutralizing activity to lethal toxin was evaluated 5 days prior to challenge (all results below the limit of detection of 0.054 NF_50_) and at days 10 and 28 post-challenge in GEP-treated animals. All saline-treated control animals (*n* = 6) succumbed between days 2 and 5 post-challenge.

Prior to challenge, no toxin neutralization assay (TNA) neutralizing titers were detected in any animals. Neutralization factor-50 (NF_50_) titers were detected only in gepotidacin-treated animals that survived beyond day 5. By day 10 post-challenge, all surviving animals had NF_50_ titers ranging from 0.23 to 9.5. At study termination (day 28 post-challenge), NF_50_ values increased and ranged from 1.55 to 17.5 (Fig. 3).

### Gepotidacin is efficacious at exposures below the predicted human exposure in rabbits

**Fig 4:**
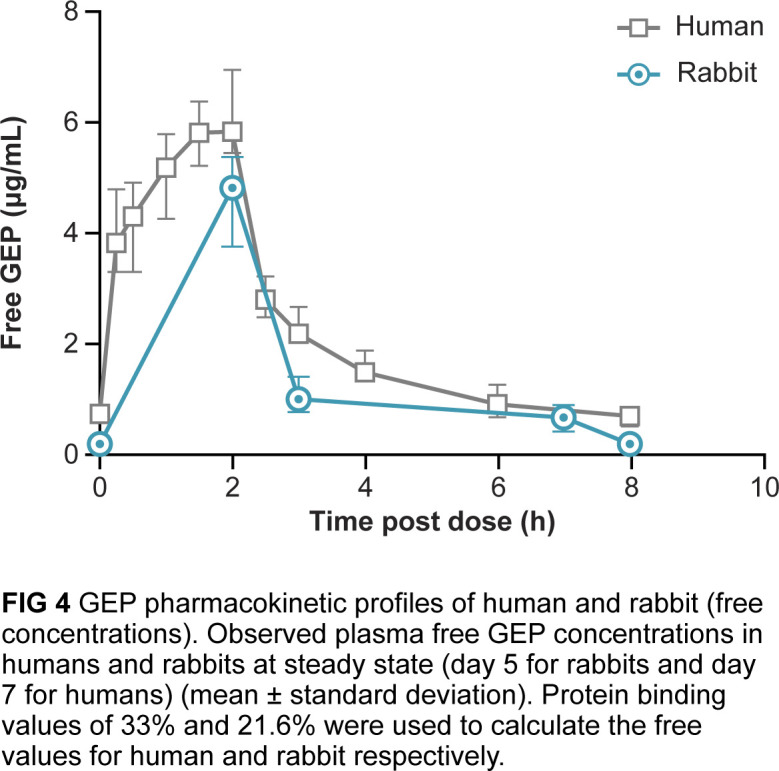
Gepotidacin (GEP) pharmacokinetic profiles of humans and rabbits (free concentrations). Observed plasma free gepotidacin concentrations in humans and rabbits at steady state (day 5 for rabbits and day 7 for humans) (mean ± standard deviation). Protein-binding values of 33% and 21.6% were used to calculate the free-drug values for human and rabbit respectively.

The administered gepotidacin dosing regimen of 114 (mg/kg/d) to rabbits provided free-drug C_max_ (*f*C_max_) and free-drug area under the concentration curve (*f*AUC) levels in plasma below exposures associated with the 1,000 mg i.v. TID human dose. Steady-state plasma pharmacokinetic profiles are shown in Fig. 4. Application of the previously determined rabbit plasma protein-binding value of 21.6% (20) and human plasma protein-binding value of 33.0% (31, 32) did not exceed *f*C_max_ in the rabbit compared to that in humans, *f*C_max_ (4.7 and 6.1 µg/mL, respectively), and the time to peak plasma concentration (T_max_) was 2 h in both rabbits and humans. The 24-h *f*AUC in the rabbit was also below the human *f*AUC (33 and 57 µg·h/mL, respectively).

### Gepotidacin treatment reduces the tissue bacterial burden and damage associated with *B. anthracis* infection

At necropsy, gross lesions were evident in three of six saline control animals that died or were euthanized due to anthrax. Gross lesions included enlarged mediastinal lymph nodes, with clear fluid in the abdominal, thoracic, and/or pericardial cavity, and red discoloration of the meninges. No gross lesions were seen in gepotidacin-treated rabbits, including the rabbit that succumbed to anthrax on day 3.

**Fig 5:**
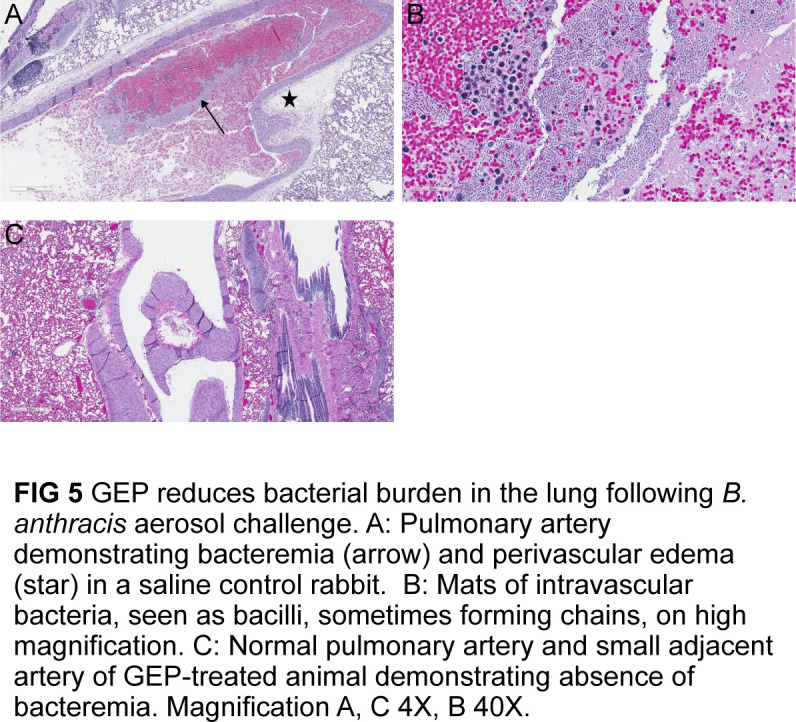
Gepotidacin reduced bacterial burden in the lungs following *B. anthracis* aerosol challenge. (**A**) Pulmonary artery demonstrating bacteremia (arrow) and perivascular edema (star) in the saline control rabbit. (**B**) Mats of intravascular bacteria, seen as bacilli, sometimes forming chains, on high magnification. (**C**) Normal pulmonary artery and small adjacent artery of gepotidacin-treated animals demonstrating absence of bacteremia. Magnification A and C, 4X; B, 40X.

Microscopy findings of control animals and of the single gepotidacin-treated animal that succumbed were consistent with published reports of anthrax in rabbits (2). In these animals, there was fibrinous pneumonia with edema and intravascular and extravascular bacteria (Fig. 5). Intra- and/or extravascular bacteria were also identified in the meninges, brain, liver, spleen, and mediastinal lymph nodes, often associated with hemorrhage and necrosis.

## DISCUSSION

Inhalational anthrax is a disease mediated by bacterial growth and toxemia, leading to inflammation, tissue damage, and, most often, acute death. In this study, gepotidacin, a novel, bactericidal, triazaacenaphthylene antibacterial rapidly cleared inhaled *B. anthracis* Ames strain from the blood and tissues of rabbits, reducing fever, inflammation, and subsequent tissue damage, resulting in enhanced survival. Animals that completed the gepotidacin dosing regimen survived until study end, whereas all saline controls succumbed to anthrax. Gepotidacin is widely distributed in tissues following i.v. infusion, with an alveolar macrophage:unbound plasma AUC ratio of 178:1(33). Once inside the lungs, anthrax spores are engulfed by alveolar macrophages and transported to mediastinal and peribronchial lymph nodes where the spores may germinate. Given the importance of macrophages in the progression of infection (1, 6), the high concentrations of gepotidacin in the macrophages could be beneficial in terms of cell killing once the spores have germinated, potentially limiting progression of the disease. Ionin et al. recently described a TNA NF_50_ threshold predictive of immunity to LT and survival of rabbits following anthrax vaccination with subsequent re-challenge (30). In those studies, a TNA NF_50_ titer of 0.56 corresponded to a 70% probability of survival in rabbits following re-challenge. In the current study, all gepotidacin-treated survivors assessed had an NF_50_ titer greater than 0.56 (1.5–17.5) on day 28 post-challenge. These data suggest that following treatment of an active infection with gepotidacin, surviving rabbits may mount an immune response that would enhance survival following exposure to a new aerosol challenge or potential germination of residual spores. In addition, gepotidacin demonstrated *in vitro* activity against diverse sets of *B. anthracis* isolates, suggesting gepotidacin would provide coverage against isolates that may be encountered in the real world.

One of the therapeutic challenges of anthrax infection is that it is the spore, rather than the vegetative cell, that is the infectious agent, and residual spores in the body may germinate after cessation of antibiotic therapy, producing an active infection. Following inhalational challenge, spores are phagocytosed by resident alveolar macrophages and dendritic cells and transported via the circulatory system to the mediastinal and peribronchial lymph nodes where they germinate. In this study, consistent with the hematologic dissemination from the lungs, intravascular bacteria were found in distal organs, such as the spleen, liver, and brain, of saline-treated controls. All animals had minimal to mild histiocytic cellular infiltrates within the lungs, indicative of an immune response to infection; however, there were no microscopic lesions consistent with anthrax in the gepotidacin-treated animals.

Steady-state plasma concentrations for rabbits given a dosing regimen designed to achieve plasma exposure profiles that simulate the human 1,000 mg i.v. TID regimen demonstrated C_max_ and AUC values below those seen clinically in humans, demonstrating that the 1,000 mg i.v. TID regimen achieves gepotidacin exposures shown to be efficacious in these pivotal Animal Rule studies.

The FDA Animal Rule provides the basis to establish efficacy in support of human treatment, using a well-characterized animal efficacy model, in cases where it is not practical or ethical to conduct testing on humans (26, 27). The NZW rabbit model of inhalational anthrax utilized for this study has been shown to be an appropriate model for disease pathogenesis in humans. Furthermore, the similarities of the rabbit model to human disease make it suitable for evaluation of antibacterial therapies. The study presented here was blinded and randomized and designed to provide pivotal Animal Rule efficacy data for use of gepotidacin for anthrax treatment. *In vitro* studies against diverse collections of *B. anthracis* demonstrated no pre-existing gepotidacin resistance, supporting gepotidacin as a potential treatment option against anthrax strains resistant to standard antibiotic classes. The combination of the novel mode of action, *in vitro* activity, a favorable pharmacodynamic profile, and efficacy in the rabbit anthrax model supports gepotidacin as a potential treatment for anthrax, including infections caused by antibiotic-resistant strains.

## MATERIALS AND METHODS

### *B. anthracis* isolate collections and MIC testing

MICs were determined by the broth microdilution method on 130 isolates at USAMRIID, including at least 17 genotypes from a diverse geographic distribution (34). Thirty additional isolates were tested from the culture collection at Rutgers University. MICs were also determined against the attenuated (plasmid-cured ΔANR) *B. anthracis* Ames strain and two single-step DNA gyrase mutant isolates at USAMRIID (35, 36). All testing was conducted according to Clinical and Laboratory Standards Institute (CLSI) guidelines (37, 38). Gepotidacin MICs were determined for the *in vivo B. anthracis* Ames challenge strain and on positive cultures from terminal blood or tissue sample plates collected from gepotidacin-treated animals. Three to five colonies were assayed in triplicate from the same inoculum.

### Circulating PA levels (PA-ECL)

The presence of *B. anthracis* PA suggests an active anthrax infection (28, 30, 39) and has been correlated with bacteremia. Prior to the challenge, and at 6-h intervals beginning 24 h post-challenge until 72 h post-challenge, 1 mL of blood was collected in serum separator tubes, and an aliquot of the serum was evaluated in the PA-ECL assay. A positive PA-ECL result was used as the trigger for treatment (29, 40).

### Toxin neutralization assay

The TNA is designed to measure the functional ability of serum antibodies to neutralize *B. anthracis* lethal toxin activity. The assay was performed as previously described using an *in vitro* cytotoxicity assay from the serum sampled, as outlined in Fig. 3 (41).

### Preparation of test article and dose formula analysis

Gepotidacin was dissolved in 0.9% sterile saline (pH 5.0 to 5.5) and filter-sterilized. All dosing solutions were prepared daily and maintained at ambient temperature for infusion. Aliquots from gepotidacin formulations were analyzed on the day of preparation to confirm the concentration of gepotidacin in the dose solution prior to use in the study.

### *In vivo* NZW rabbit model of inhalational anthrax

All animal procedures were approved by the GSK’s and Battelle’s (a contract research laboratory with expertise in FDA Animal Rule studies) Institutional Animal Care and Use Committee and the U.S. Army Medical Research and Materiel Command’s Animal Care and Use Review Office and were conducted in an Association for Accreditation and Assessment Laboratory Animal Care-accredited facility in compliance with U.S. regulations governing the housing and use of animals. This study was conducted in coordination with the FDA and was completed under the agency’s Special Protocol Assessment process. All exposures and assays were performed in a Biosafety Level-3 laboratory registered and approved with the Centers for Disease Control and Prevention (CDC) and inspected by the U.S. Departments of Defense and Agriculture. The Battelle CDC principal investigator approved the use of *B. anthracis* on rabbits in this study. In addition, this study was conducted in compliance and reviewed by the Chair of Battelle’s Biological Safety Committee, which has oversight of all risk group 2 and 3 biological research at Battelle. All studies were conducted humanely and complied with national laws, guidelines, and company policies for the care, welfare, and treatment of animals.

### Test system

Male and female NZW rabbits (*Oryctolagus cuniculus*) weighing at least 3 kg were obtained from Covance (Denver, PA), a U.S. Department of Agriculture-licensed facility. All rabbits were confirmed negative for prior exposure to *B. anthracis* using the TNA and for the presence of an active *B. anthracis* infection using the PA-ECL assay prior to challenge. Rabbits were housed individually, in stainless steel cages, on racks equipped with automatic watering systems. Rabbits were surgically implanted with dual cath-in-cath vascular access ports (VAPs) for antibacterial dosing and for blood collection. A tether was attached to the jacket and to a swivel in the top of the cage to allow for free movement of the animal without twisting the infusion line. Rabbits were observed for clinical signs either twice daily [BID (before challenge and from day 7 through day 28 post-challenge)] or four times daily [QID (from day 1 to day 7 post-challenge)]. Any animal judged to be moribund was humanely euthanized.

### Surgical procedures

Prior to animal arrival, two VAPs were surgically implanted into rabbits by the vendor, one in the jugular vein and the other in the femoral vein. The port from the jugular vein catheter was utilized for blood sampling, and the port from the femoral vein catheter was used for dosing.

### Body temperature

Rabbits were sedated with acepromazine (1 to 5 mg/kg, intramuscularly), and transponders (IPTT-300, BMDS, Seaford, DE) were injected subcutaneously into the rump of each rabbit. Mean baseline temperatures were established for each rabbit from day 5 until just prior to challenge. Following challenge, temperatures were recorded hourly for the first 3 days post-challenge and BID from day 4 post-challenge through study termination (day 28 post-challenge).

### Inhalational challenge

Rabbits were exposed (muzzle only) to an aerosolized dose of *B. anthracis* spores, targeting 200 × LD_50_s (2.1 × 10^7^ spores) by real-time plethysmography (42). Post-exposure aerosol concentrations of *B. anthracis* were quantified by determination of the CFU from the effluent streams of the animal exposure port by an in-line impinger (Model 7541, Ace Glass Incorporated, Vineland, NJ) (29). The gepotidacin MIC for the Ames *in vivo* challenge strain determined at Battelle was 2 µg/mL.

### Randomization

Animals were randomized to the treatment group at the time of a positive PA-ECL result (trigger).

### Test article administration

Intravenous infusions of gepotidacin or vehicle (0.9% saline) were administered based on a positive serum PA result in the ECL assay (trigger for treatment). The first treatment was initiated within 3 to 4.3 h (mean 3.8 h) of a documented positive ECL result. Gepotidacin dose or a corresponding volume of the saline vehicle was based on the individual body weights collected on the day of aerosol exposure. Gepotidacin was administered as a 2-h infusion (30 mg/kg), followed 1 h later by a 4-h infusion of 8 mg/kg. This dose regimen was repeated TID every 24 h, for a total daily dose of 114 mg/kg (six total infusions). Treatment was continued for 5 consecutive days. Doses were designed to simulate plasma exposures consistent with a human 1,000 mg i.v. TID dose in terms of the shape of the exposure profile and daily *f*AUC values.

### Gepotidacin plasma analysis

On treatment days 1 and 5, blood samples were collected prior to treatment (PTT) and at 2 h, 3 h, 7 h, and 8 h after start of treatment. Blood samples collected from the VAP were chilled, centrifuged for plasma processing, filtered, tested for sterility, and maintained at ≤–70°C until assayed. Gepotidacin concentrations in plasma were analyzed using ultra-high performance liquid chromatography (UHPLC) with tandem mass spectrometry (MS/MS) detection. T_max_ was observed at 2 h after the start of the first and 25th infusion for all animals. There was no marked (>2 fold) difference in systemic exposure (mean C_max_ and AUC0-t values) between females and males after either the first or 25th infusions. Free gepotidacin pharmacokinetic analysis results were based on steady-state sampling on day 5 for rabbits and day 7 for humans.

### Human pharmacokinetics

The pharmacokinetics of gepotidacin following repeat i.v. dosing were previously determined in healthy human volunteers in accordance with the International Conference on Harmonization-Good Clinical Practice guidelines and applicable subject privacy requirements and guidelines (43). Eight-hour dosing intervals were maintained for TID regimens.

### Bacteriology assessments

At predetermined time points, whole blood was collected in sodium polyanethol sulfonate tubes and was assessed quantitatively for the presence of bacteria with colony morphology consistent with that of *B. anthracis*. Following necropsy and prior to fixation, ~1 cm^3^ samples of the heart, brain, lung, kidney, spleen, lymph nodes, and gross lesions from each animal were aseptically collected, weighed, homogenized, and cultured by spread plate enumeration and assessed quantitatively for colony morphology consistent with that of *B. anthracis*. The bacterial burden was reported as CFU/g tissue or CFU/mL of blood. Gepotidacin MICs were determined on any isolates recovered from blood and/or tissues of gepotidacin-treated animals by selecting three to five colonies and testing MICs in triplicate from the same inoculum.

### Necropsy/histopathology

A complete necropsy was performed on all study animals found dead, euthanized in moribund condition, or at study termination. Gross necropsies included examinations of the external surfaces of the body; all orifices; and the cranial, thoracic, and abdominal cavities and their contents. Target tissues including those of the brain, lungs, liver, spleen, and mediastinal lymph nodes along with gross lesions were collected, preserved in 10% neutral-buffered formalin, stained with hematoxylin and eosin, and examined microscopically to confirm death or illness due to anthrax, by a study pathologist who was blinded to the treatment group.

### Statistical analyses

Boschloo’s tests were conducted in *R* (version 3.3.1 64-bit). For a one-sided test, the 0.025 level was considered significant. Analyses were also conducted with SAS (version 9.4 64-bit). Other analyses were conducted using GraphPad Prism version 7.03, where a *P-*value of <0.05 was considered statistically significant.

## Data Availability

Data associated with this study are presented in the paper. Requests for materials will be reasonably considered, and such requests should be addressed to the corresponding author.
